# Direct Nanoscale Visualization of the Electric-Field-Induced Aging Dynamics of MAPbI_3_ Thin Films

**DOI:** 10.3390/ma16124277

**Published:** 2023-06-09

**Authors:** Nikita A. Emelianov, Victoria V. Ozerova, Yuri S. Fedotov, Mikhail V. Zhidkov, Rasim R. Saifutyarov, Maria S. Malozovskaya, Mikhail S. Leshchev, Eugeniy V. Golosov, Lyubov A. Frolova, Pavel A. Troshin

**Affiliations:** 1 Federal Research Center of Problems of Chemical Physics and Medicinal Chemistry, Russian Academy of Sciences, Academician Semenov Ave. 1, Chernogolovka 142432, Russia; emelianov@icp.ac.ru (N.A.E.);; 2 Institute of Solid State Physics, Russian Academy of Sciences, Academician Osipyan Str. 2, Chernogolovka 142432, Russia; 3 National Research Centre “Kurchatov Institute”, Moscow 123182, Russia; 4 Harbin Institute of Technology (HIT), 92 West Dazhi Street, Nan Gang District, Harbin 150001, China; 5 Zhengzhou Research Institute of HIT, 26 Longyuan East 7th, Jinshui District, Zhengzhou 450000, China

**Keywords:** methylammonium lead iodide (MAPbI_3_), field-induced degradation, infrared scattering-type scanning near-field microscopy (IR s-SNOM), photoluminescence (PL) microscopy, confocal microscopy, scanning electron microscopy (SEM), energy-dispersive X-ray (EDX) microanalysis, time-of-flight secondary ion mass spectrometry (ToF-SIMS)

## Abstract

Perovskite solar cells represent the most attractive emerging photovoltaic technology, but their practical implementation is limited by solar cell devices’ low levels of operational stability. The electric field represents one of the key stress factors leading to the fast degradation of perovskite solar cells. To mitigate this issue, one must gain a deep mechanistic understanding of the perovskite aging pathways associated with the action of the electric field. Since degradation processes are spatially heterogeneous, the behaviors of perovskite films under an applied electric field should be visualized with nanoscale resolution. Herein, we report a direct nanoscale visualization of methylammonium (MA^+^) cation dynamics in methylammonium lead iodide (MAPbI_3_) films during field-induced degradation, using infrared scattering-type scanning near-field microscopy (IR s-SNOM). The obtained data reveal that the major aging pathways are related to the anodic oxidation of I^−^ and the cathodic reduction of MA^+^, which finally result in the depletion of organic species in the channel of the device and the formation of Pb. This conclusion was supported by a set of complementary techniques such as time-of-flight secondary ion mass spectrometry (ToF-SIMS), photoluminescence (PL) microscopy, scanning electron microscopy (SEM) and energy-dispersive X-ray (EDX) microanalysis. The obtained results demonstrate that IR s-SNOM represents a powerful technique for studying the spatially resolved field-induced degradation dynamics of hybrid perovskite absorbers and the identification of more promising materials resistant to the electric field.

## 1. Introduction

Hybrid materials based on lead halides with a perovskite crystal structure currently appear to be the most promising emerging technology in the new generation of solar cells. In addition to their power conversion efficiency, which in recent years has reached impressive values (of up to 25.8%) [1] comparable to the best silicon photovoltaic cells, devices based on perovskite-type semiconductors have significantly lower cell manufacturing costs. In addition, they have a number of advantages related to mass–size characteristics and can also be produced on flexible substrates. At the same time, the bottleneck leading up to their successful commercialization is low operational stability, which is due to a set of stress factors of external and internal natures [2]. Among the external stress factors, the effects of atmospheric moisture and oxygen are the most important. Thus, the encapsulation of the active layer can be used to overcome these issues. On the contrary, the internal factors associated with the processes occurring in perovskite films upon device operation, such as photo-, thermal-, and field-induced degradation, need to be studied thoroughly in order to gain control over the intrinsic stability of lead halide perovskite solar cells.

It is known that the thermal degradation of MAPbI_3_ and MAPbI_3-x_Cl_x_, where MA is a methylammonium CH_3_NH_3_^+^ cation, results in the formation of CH_3_I, NH_3_, CH_3_NH_2_ and HI as reaction products [3]. The light-induced decomposition of MAPbI_3_ induces the formation of a wide range of organic species, in addition to metallic lead and molecular iodine. For complex lead halides with different monovalent cations and halide anions, similar aging pathways have been demonstrated [4].

Under the real operational conditions, the active layers of solar cells are not only exposed to light and increased temperatures but also have to tolerate the electric field arising from the differences in the work functions of the electrodes (built-in potential) and due to the photoelectric effect (photopotential). Charges are generated and transferred in the photoactive layer, and their physical carriers are cations or anions, which are at least partially localised on the molecular fragments of the perovskite lattice. The trapping of the charges results in their further localization, which could lead to the disintegration of the perovskite framework with the formation of different products, e.g., I_2_/I_3_^−^ due to hole trapping and Pb^0^ due to electron trapping [5]. Thus, the electrochemical degradation of the photoactive layer can play a crucial role in defining the lifetime of a perovskite solar cell [6].

It should be noted that the processes occurring during field-induced degradation have been studied less compared to photothermal degradation. For the benchmark photoactive perovskite material MAPbI_3_, it was shown that an applied electric field causes the anodic oxidation of I^−^ to I_2_, which remains in the film in the form of polyiodides; hence, the process can be reversed upon reduction (i.e., applying a bias of opposite polarity) [6]. In contrast, the reduction of the organic methylammonium cation produces volatile products that leave the film and thus make the degradation irreversible.

It was also shown that applying the bias to the gold/MAPbI_3_ interface results in gold oxidation and the further transport of Au^+^ ions towards the bulk of the active layer [7]. Replacing gold with ITO or FTO electrodes suppresses this type of degradation but causes a number of other problems related to the contact resistance of the electrode/perovskite interface, irreversible strain effects and interfacial atomic mixing at MAPbI_3_/ITO junction under the influence of the applied electric bias [8]. Formamidinium (FA), guanidinium (GA) and cesium (Cs) cations and their combinations generally enable higher photochemical and thermal stabilities of complex lead halides when compared to methylammonium [9,10]. Therefore, an enhanced electrochemical stability could also be expected for lead halide perovskites with FA, GA and Cs cations, though this subject still requires a thorough exploration. Thus, for the formamidinium lead iodide, FAPbI_3_, ToF-SIMS visualized the degradation of the perovskite/gold electrode under the action of an electric field [11]. Interestingly, it was shown that for FA_0.95_Cs_0.05_Pb(I_0.83_Br_0.17_)_3_, using gold as an electrode prevents perovskite degradation due to the formation of some complexes with dopants in a PTAA-based hole transport layer [12]. It was also shown that the main degradation products are PbI_2_ and δ-FAPbI_3_. 

The bias behaviors of Cs_0.05_FA_0.85_MA_0.10_Pb(I_0.85_Br_0.15_)_3_-based devices were also investigated, and the lead iodide PbI_2_ was found to be the main product of the perovskite field-induced degradation. The use of depth-resolved XPS/UPS/REELS allowed for the determination of electrically passive trace amounts of PbI_2_ and I_2_ everywhere, moving from the top Cu electrode to the perovskite surface [13]. No evidence of electrode corrosion by I_2_ to form CuI at the Cu/organic interface was found. This highlights the importance of using transport and buffer layers to stabilize perovskite/electrode interfaces [14,15]. 

Thus, the processes of ion migration in perovskite films upon the influence of an electric field seriously hinder the further development and large-scale commercialized production of perovskite solar cells [16]. Therefore, studying the decomposition dynamics of perovskites in an electric field is of considerable practical interest, both in terms of identifying the most stable materials and in investigating other approaches to stabilization, such as the modification of perovskite/electrode interfaces. Infrared spectroscopy has been shown to be a powerful technique for studying the degradation of perovskite materials under various stress factors, including environmental conditions and photo-irradiation [17]. To study the organic cation dynamics with nanometer spatial resolution in the course of absorber material photodegradation, we previously successfully used the IR s-SNOM technique [18]. Herein, we present the results of a systematic experimental study of the field-induced degradation dynamics of MAPbI_3_ films using a set of complementary techniques, including a combination of the IR s-SNOM technique with confocal and PL-microscopy, SEM+EDX and ToF-SIMS.

## 2. Materials and Methods

All the solvents and reagents for the fabrication of perovskite films were purchased from Sigma-Aldrich (St. Louis, MO, USA) or Acros Organics (Geel, Belgium) and used as received or purified according to the standard procedures. PbI_2_ (99.9%) was purchased from LANHIT (Moscow, Russia). N,N-Dimethylformamide (DMF) (99.8%) and toluene (99.8%) were received from ACROS (Fukuoka, Japan). Methylammonium iodide (MAI, CH_3_NH_3_I) was the product of FOMaterials Ltd. (Chernogolovka, Russia). The glass substrates were cleaned sequentially with toluene and acetone and then subjected to RF plasma treatment (air plasma, base pressure 10^−2^ mbar, 150 W) for 5 min. The perovskite films were grown on glass substrates via spin coating the 1.35 M MAPbI_3_ precursor solution, which was based on an equimolar mixture of PbI_2_ and MAI in anhydrous DMF. A 45 μL aliquot of the precursor solution was dropped onto the glass substrate rotating at 3000 rpm and then quenched after 7–8 s by dripping 120 μL of anhydrous toluene. The sample was kept at 3000 rpm for an additional 20 s and then annealed at 100 °C for 5 min [19]. All procedures were carried out in an inert atmosphere (O_2_ < 0.1 ppm, H_2_O < 1 ppm) inside an MBraun glove box. The typical thickness of the perovskite films, determined from cross-sectional scanning electron microscopy images, was about 400 nm. Gold electrodes were thermally evaporated on top of the perovskite films via a shadow mask in a vacuum chamber integrated in the glove box at a base pressure of 6 × 10^−6^ mbar. The channel lengths in the produced lateral resistor devices were 50 µm for samples prepared for IR s-SNOM, confocal microscopy and SEM/EDX measurements and 200 µm for the samples used for PL microscopy and ToF-SIMS measurements. The samples were polarized using Keithley 2400 source meters (Keithley Instruments, Solon, OH, USA) that generated a stabilized electric bias voltage of 1 V/µm. 

The IR s-SNOM measurements were carried out using a neaSNOM microscope (Neaspec, Haar, Germany) in PsHet mode with a Mid-IR laser MIRcat-2400 (Daylight Solutions, San Diego, CA, USA) inside the MBraun glove box. The polarized channel and electrode edges of an 80 × 20 µm^2^ region were scanned in 200 × 50 pixels at the speed of 7–8 µm/s using ARROW-NCPt (NANOWORLD, Neuchâtel, Switzerland) cantilevers with PtIr coatings with a probe radius of <25 nm, with a typical resonance frequency of 285 kHz and a stiffness of 42 N m^−1^. The cantilever oscillation amplitude reached 50–65 nm. The scans were performed at the IR frequency of 1249 cm^−1^, which corresponds to NH_3_ rocking vibrations in a methylammonium cation [18]. The previously developed techniques were used to suppress image artifacts caused by the indirect illumination of the probe due to far-field reflection effects from the sample surface [20]. The images presented below corresponding to the absorption intensities of the IR signal were basically obtained as the phase difference of the 3rd and 2nd harmonics, φ_3_ − φ_2_. Mathematical processing of the measurement results was carried out in Gwyddion 2.58 [21].

The PL microscopy images with a total area of 400 × 400 µm^2^ and a pixel size of 5 × 5 µm^2^ were obtained for the samples placed in a sealed quartz cell using a DRX-2 microscope (Thermo Scientific, Waltham, MA, USA) with a 785 nm laser and 10× objective with an NA of 0.50.

The optical images of the channels before and after the field-induced degradation were obtained using a confocal scanning laser microscope Optelics Hybrid (Lasertec, Yokohama, Japan) with a xenon lamp and a 405 nm laser as illumination sources.

The scanning electron microscopy and X-ray microanalysis were performed with a Jeol JSM 7100F microscope (JEOL, Tokyo, Japan) and an Oxford Instrument X-Max 50 detector (Abingdon upon Thames, UK) with an accelerating voltage of 5 kV and a probe current of 350 pA. The calculated Monte Carlo locality of analysis was 420 nm.

The chemical mapping was performed using a TOF.SIMS 5-100P instrument (ION-TOF GmbH, Münster, Germany, 2007). The mapping was carried out separately for positive and negative ions due to the technique limitations. Prior to the analysis, the sample surface was cleaned over an area of 1000 × 1000 µm^2^ using a 2 keV ion beam with an exposure time of 30 s. The sputter beam was 340 nA O_2_^−^ or 120 nA Cs^+^ for the negative and positive secondary ion analysis, respectively. After the surface cleaning, 80 analysis frames, each consisting of 256 × 256 pixels, scanned over 400 × 400 µm^2^ area, were acquired in each experiment. The ion analysis consisted of Bi^+^ pulses (25 keV ion energy, 20 ns pulse duration, 3.5 pA measured sample current) and was set in spectroscopy (high-current bunched) mode with an effective target pulse width of ~1 ns. For all the interest fragments, the mass resolution was >7000 (m/δm). After data collection, the total area images were individually reconstructed and analyzed. The color scales of the reconstructed ion images were set equally between the samples separately for each species to allow a fair comparison of the secondary ion intensity.

## 3. Results

**Figure 1:**
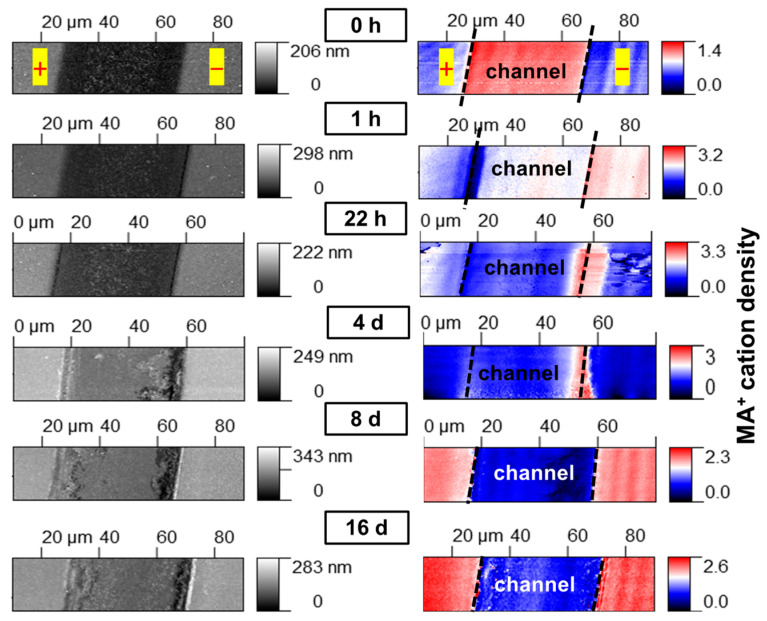
The evolution of the AFM topography (**left** column) and MA^+^ cation distribution revealed by IR s-SNOM (**right** column) in MAPbI_3_ channel between two lateral gold electrodes upon field-induced degradation. The black dashed lines in the right column should be considered a guide for the eye to show the electrode and channel boundaries.

First, we applied the IR s-SNOM technique to directly visualize the nanoscale dynamics of the MA^+^ cations upon exposure to an electric field. Previously, a similar nano-IR microscopy technique revealed the migration of organic cations toward a cathode already within 1–2 min after the exposure to an electric bias [22]; however, the aging effects have not been studied yet on a longer time scale. We herein observed a severe depletion of organic cations in the anode region 1 h after applying the bias (Figure 1). 

After 22 h, the device channel was almost entirely depleted with respect to the organic cations, which accumulated in a narrow region near the cathode and also migrated atop the electrode. After 4 days, the distribution of the cations was quite similar, while their overall amount seemed to be decreasing. The AFM topography images reveal that the perovskite/cathode interphase undergoes severe destruction with the formation of deep cavities. This observation could be considered a result of the material loss upon field-induced decomposition and concurs with the previously reported decay of methylammonium cations into gaseous reaction products [3,6]. There is also a change in the structure of the anode, apparently caused by the metallic gold oxidation and the following diffusion of Au^+^ ions into the device channel. After 8 days of field-induced degradation, the intensity of the IR signal at the frequency corresponding to the absorption of MA^+^ cations (1249 cm^−1^) became significantly weaker over the entire channel. It is worth noting that the predominant signal coming from the area of the electrodes where traces of organic species (methylamine of MA^+^) were deposited over the course of the channel area degradation. The measurements after 16 days of the sample’s exposure to the electric field delivered very similar results.

The obtained results suggest a complete loss of the organic cations from the surface of the perovskite film occurring in the device channel within 8 days of the bias exposure. However, it is known that the signal generation depth in IR s-SNOM is about several tip radii [22]. Thus, since the perovskite film thickness in the studied samples was approximately 400 nm, some cations could be potentially preserved in the depth of the film since the electric field strength was slightly weaker when compared to the film surface.

**Figure 2:**
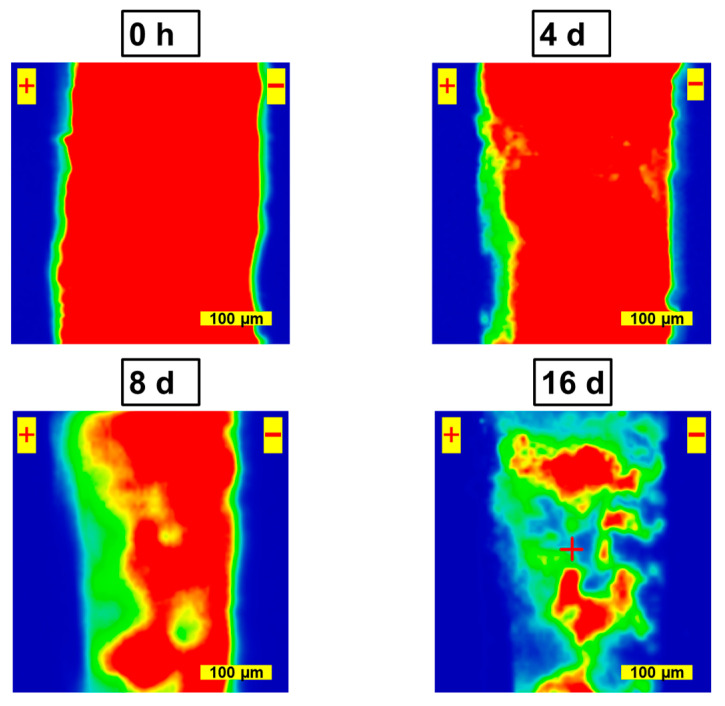
PL microscopy images of MAPbI_3_ channel before and after 4, 8 and 16 days of bias exposure.

PL microscopy is a sensitive method for detecting defects in perovskites. Herein, we applied this technique to trace the changes in the photoluminescence of the perovskite films over a 200 µm channel upon field-induced degradation (Figure 2). 

After 4 days of field-induced degradation, an intense quenching of luminescence near the anode was observed. The migration of I^−^ ions toward the anode in the applied electric field, followed by their oxidation to molecular I_2_ (which could be trapped as I_3_^−^), leads to the formation of vacancy-type defects [4,16]. The longer exposure time led to further luminescence decay along the anode after 4 days. It should be noted that the presence of the photoluminescence signal in the cathode region after 8 days of bias exposure does not contradict the IR s-SNOM data, which suggested the complete decomposition of the perovskite in the channel. As noted above, the depth of the IR absorption signal generation in the s-SNOM method is comparable to 2–3 probe radii [23], which provides a value of 40–60 nm. At the same time, the penetration depth of the photoluminescence excitation beam for MAPbI_3_ exceeds 2 µm [24]. Thus, the source of the photoluminescence could be located deeper within the perovskite film. Another explanation could be based on the reasonable assumption that the devices with relatively short channels (50 µm) used for IR s-SNOM measurements degrade faster than the devices with longer channels required for PL mapping (both techniques could not be applied to the channels of the same size due to instrumental limitations). 

After 16 days, an almost complete quenching of the luminescence in the channel was observed. It is worth noting that the maximal PL intensity was finally observed in the middle of the device channel, which is consistent with the fact that material degradation occurs at both electrodes. IR s-SNOM is very sensitive to organic cations; hence, it can visualize their degradation at a cathode. On the contrary, the anodic oxidation of iodide anions is not clearly manifested in IR spectra but could be clearly visualized by PL microscopy. Thus, IR s-SNOM and PL microscopy deliver complementary information which is essential for understanding the field-induced degradation of the perovskite films.

**Figure 3:**
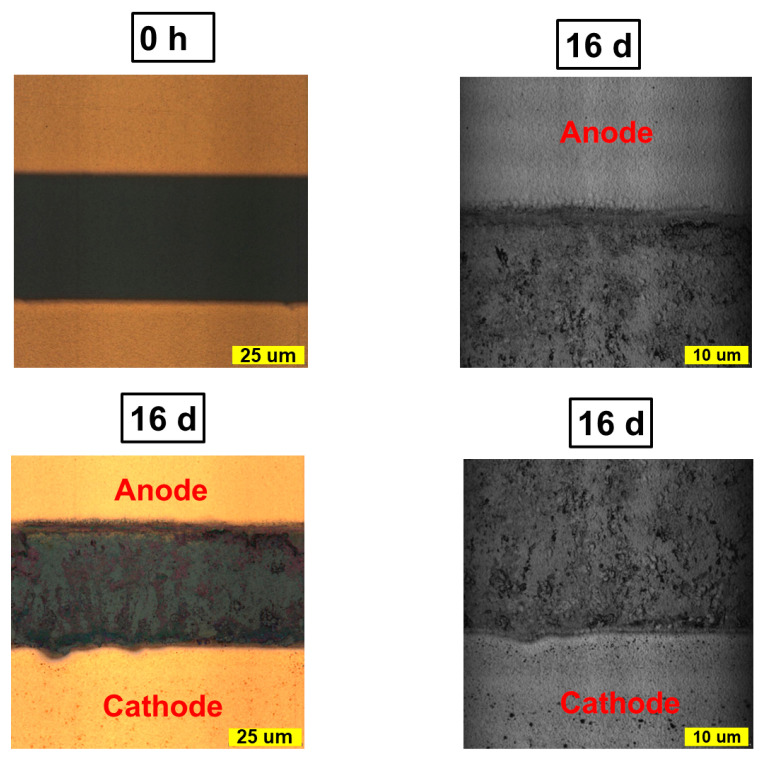
Confocal microscopy of MAPbI_3_ channel before and after exposure to electric bias for 16 days (illumination source: xenon lamp) (**left** column). Zoomed-in parts of the anode and cathode after bias exposure are shown for clarity (illumination source: 405 nm laser) (**right** column).

The confocal microscopy images of the channel demonstrate significant changes after the bias exposure (Figure 3). The yellow metallic shadow observed in the channel at the anode/perovskite interphase suggests the diffusion of gold into the channel upon bias exposure. Erosion of the active material occurs at both the cathode and the anode, which corroborates with the IR s-SNOM and PL microscopy data evidencing the cathodic reduction of methylammonium and the anodic oxidation of iodide. In addition, the formation of large and poorly ordered grains of aging products approaching several micrometers in size was observed on the entire surface of the channel.

**Figure 4:**
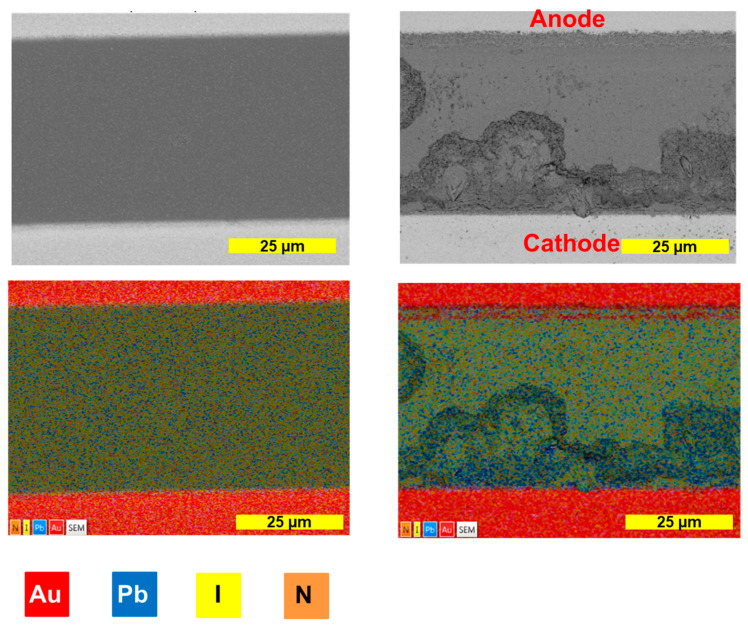
SEM microscopy and EDX mapping (red—Au; blue—Pb; yellow—I; orange—N) of MAPbI_3_ channel before (**left** column) and after (**right** column) bias exposure for 16 days.

The SEM images also confirm the erosion of the near-cathode region of the channel, with the formation of caverns as a result of the perovskite decomposition under the action of the bias voltage (Figure 4). Erosion of the anode region is also observed, but to a lesser extent. The aging products, as follows from the previous reports and our results, could be represented by PbI_2_ or metallic lead. The intense X-ray emission of lead from the cavern area near the cathode with the absence of a colocalized iodide emission in these areas testifies in favor of the formation of lead as the final product of the field-induced degradation of MAPbI_3_ (Figure 4). EDX mapping also confirms the diffusion of gold from the anode to the channel. It should also be noted that the formation of the caverns near the cathode deteriorates the film uniformity, so we could detect Si, Na and O signals from the glass substrate in the EDX spectra of the aged samples (Figure S1).

**Figure 5:**
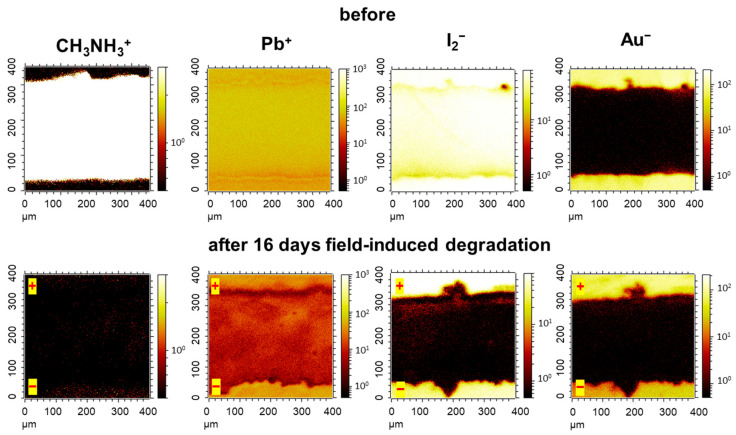
ToF-SIMS chemical maps showing the distribution of the characteristic marker ions CH_3_NH_3_^+^, Pb+, I_2_^−^ and Au^−^ in the fresh MAPbI_3_ channel and after 16 days of field-induced degradation.

Another powerful technique for extracting information on the local chemical composition of the sample is time-of-flight secondary ion mass spectrometry (ToF-SIMS). Indeed, the ToF-SIMS mapping of the aged sample revealed a complete loss of the organic cations from the perovskite film in the device channel after 16 days of bias exposure, which perfectly matches the IR s-SNOM data (Figure 5). The reduced yield of lead ions from the aged device channel when compared to the pristine one is most likely due to the partial reduction of Pb^2+^ to Pb^(0)^. The iodide (I_2_^−^ marker ions) was severely depleted in the device channel, especially at the anodic side due to I^−^ oxidation. The remaining traces of iodine in the device channel surprisingly colocalized with gold, which suggests that the anodic oxidation of Au produces some rather stable iodide species. The clear presence of lead in the channel with severely depleted iodide also indicates the formation of metallic lead as a product of the field-induced degradation of MAPbI_3_. 

## 4. Discussion

In this work, the IR s-SNOM technique was used for the first time to visualize the dynamics of MA^+^ cations in the course of the field-induced degradation of MAPbI_3_ films. 

The application of this technique allowed us to reveal a complete loss of methylammonium cations upon the field-induced degradation of the perovskite channel, thus confirming their electrochemical conversion to some volatile species. At the same time, PL microscopy provided complementary information, in particular revealing the luminescence quenching progressing from the anode side, which is a signature of I^−^ oxidation and the formation of vacancies and I_3_^−^ point defects. It is worthy to note that PL quenching begins from the anode, where methylammonium cations are displaced due to the action of the electric field. Therefore, the depletion of organic cations depletion near to the anode could contribute to the observed PL quenching and probably also facilitates the anodic I^−^ oxidation. 

It is known that the cathodic reduction of CH_3_NH_3_^+^ cations results in the release of gaseous reaction products. This leads to the erosion of the material and the destruction of the perovskite/cathode interface with the formation of caverns. Similar erosion also occurs at the anode side due to the oxidation of iodide and its loss in the form of molecular iodine. Atomic force microscopy, confocal and scanning electron microscopy techniques support these findings.

Importantly, the EDX microanalysis demonstrates the presence of a substantial amount of lead in the device channel, particularly along the cathode, which is not colocalized with the iodide emission. Similar results were also obtained using ToF-SIMS, which revealed a moderate decrease in the yield of the lead marker ions after the bias exposure of the device channel, while the iodide species were severely (virtually completely) depleted. These findings strongly suggest the formation of metallic lead as the major solid product of the electrochemical degradation of MAPbI_3_. Lead iodide PbI_2_ is supposed to be an intermediate product, although we did not detect it experimentally.

Finally, we revealed the anodic oxidation of gold and its diffusion to the device channel. The colocalization of gold with iodine revealed by ToF-SIMS may suggest the formation of some stable Au iodides in the system.

Thus, the overall set of the experimental results allows us to conclude that the following processes occur upon the field-induced degradation of MAPbI_3_:

(1) The migration of methylammonium cations to the cathode and their subsequent decomposition with the release of gaseous reaction products, as reported previously [6]:
  2CH_3_NH_3_^+^ + 2e^−^ → 2CH_3_NH_2_(g) + H_2_(g) (1)
(2) The cathodic reduction of Pb^2+^ with the formation of metallic lead, which could grow in the form of dendrites through the device channel (the disordered phase of aging products revealed by confocal microscopy):
  Pb^2+^ + 2e^−^ → Pb^0^ (2)
(3) The migration of iodide anions toward the anode and their oxidation with the formation of molecular iodine, which could be partially trapped in the form of polyiodides in the initial stages of the process:
  2I^−^ − 2e^−^ → I_2_ (3)
(4) The anodic oxidation of gold and its diffusion to the device channel:
  Au^0^ − e^−^ → Au^+^ (4)

## 5. Conclusions

We have performed a systematic study of MAPbI_3_ field-induced degradation using a set of complementary methods, including IR s-SNOM, PL microscopy, confocal microscopy, SEM+EDX and ToF-SIMS. For the first time, the IR s-SNOM technique was applied to study the nanoscale dynamics of organic cations in the course of the field-induced perovskite degradation process. The obtained results not only enabled the high-resolution visualization of the dynamics of CH_3_NH_3_^+^ cations and I^−^ anions in the perovskite film upon bias exposure but also revealed associated structural and morphological changes originating from the loss of the material involved in electrochemical reactions at both a cathode and an anode. A comparison of the SEM+EDX and ToF-SIMS data allowed us to conclude that the final solid product of the field-induced degradation, MAPbI_3_, is metallic lead, which could be formed as a result of the further decay of the PbI_2_ intermediate in the near-cathode area. 

## Data Availability

Not applicable.

## Supplementary Materials

The following supporting information can be downloaded at: https://www.mdpi.com/article/10.3390/ma16124277/s1, Figure S1: SEM microscopy + EDX mapping of Si, Na, O in MAPbI_3_ channel after field-induced degradation over 16 days.

## References

[1] Best Research-Cell Efficiency Chart. https://www.nrel.gov/pv/cell-efficiency.html.

[2] Fu W., Ricciardulli A.G., Akkerman Q.A., John R.A., Tavakoli M.M., Essig S., Kovalenko M.V., Saliba M. (2022). Stability of perovskite materials and devices. Mater. Today.

[3] Spalla M., Perrin L., Planes E., Matheron M., Berson S., Flandin L. (2020). Effect of the Hole Transporting/Active Layer Interface on the Perovskite Solar Cell Stability. ACS Appl. Energy Mater..

[4] Akbulatov A.F., Luchkin S.Y., Frolova L.A., Dremova N.N., Gerasimov K.L., Zhidkov I.S., Anokhin D.V., Kurmaev E.Z., Stevenson K., Troshin P.A. (2017). Probing the Intrinsic Thermal and Photochemical Stability of Hybrid and Inorganic Lead Halide Perovskites. J. Phys. Chem. Lett..

[5] Frolova L.A., Luchkin S.Y., Lekina Y., Gutsev L.G., Tsarev S.A., Zhidkov I.S., Kurmaev E.Z., Shen Z.X., Stevenson K.J., Aldoshin S.M. (2021). Reversible Pb^2+^/Pb^0^ and I^−^/I_3_^−^ Redox Chemistry Drives the Light-Induced Phase Segregation in All-Inorganic Mixed Halide Perovskites. Adv. Energy Mater..

[6] Yamilova O.R., Danilov A.V., Mangrulkar M., Fedotov Y.S., Luchkin S.Y., Babenko S.D., Bredikhin S.I., Aldoshin S.M., Stevenson K.J., Troshin P.A. (2020). Reduction of Methylammonium Cations as a Major Electrochemical Degradation Pathway in MAPbI_3_ Perovskite Solar Cells. J. Phys. Chem. Lett..

[7] Kerner R.A., Zhao L., Harvey S.P., Berry J.J., Schwartz J., Rand B.P. (2020). Low Threshold Voltages Electrochemically Drive Gold Migration in Halide Perovskite Devices. ACS Energy Lett..

[8] Luchkin S.Y., Akbulatov A.F., Frolova L.A., Griffin M.P., Dolocan A., Gearba R., Vanden Bout D.A., Troshin P.A., Stevenson K.J. (2017). Reversible and Irreversible Electric Field Induced Morphological and Interfacial Transformations of Hybrid Lead Iodide Perovskites. ACS Appl. Mater. Interfaces.

[9] Jiang J., Xiong M., Fan K., Bao C., Xin D., Pan Z., Fei L., Huang H., Zhou L., Yao K. (2022). Synergistic strain engineering of perovskite single crystals for highly stable and sensitive X-ray detectors with low-bias imaging and monitoring. Nat. Photonics.

[10] Barichello J., Di Girolamo D., Nonni E., Paci B., Generosi A., Kim M., Levtchenko A., Cacovich S., Di Carlo A., Matteocci F. (2023). Semi-Transparent Blade-Coated FAPbBr_3_ Perovskite Solar Cells: A Scalable Low-Temperature Manufacturing Process under Ambient Condition. Solar RRL.

[11] Drozdov M.N., Yunin P.A., Travkin V.V., Koptyaev A.I., Pakhomov G.L. (2019). Direct Imaging of Current-Induced Transformation of a Perovskite/Electrode Interface. Adv. Mater. Interfaces.

[12] Planes E., Perrin L., Matheron M., Spalla M., Berson S., Flandin L. (2021). Degradation Mechanisms in a Mixed Cations and Anions Perovskite Solar Cell: Mitigation Effect of the Gold Electrode. ACS Appl. Energy Mater..

[13] Hu J., Chen P., Luo D., Wang D., Chen N., Yang S., Fu Z., Yu M., Li L., Zhu R. (2022). Tracking the evolution of materials and interfaces in perovskite solar cells under an electric field. Commun. Mater..

[14] Lin C., Liu G., Xi X., Wang L., Wang Q., Sun Q., Li M., Zhu B., Perez de Lara D., Zai H. (2022). The Investigation of the Influence of a Cu_2_O Buffer Layer on Hole Transport Layers in MAPbI_3_-Based Perovskite Solar Cells. Materials.

[15] Omarova Z., Yerezhep D., Aldiyarov A., Tokmoldin N. (2022). In Silico Investigation of the Impact of Hole-Transport Layers on the Performance of CH_3_NH_3_SnI_3_ Perovskite Photovoltaic Cells. Crystals.

[16] Yan X., Fan W., Cheng F., Sun H., Xu C., Wang L., Kang Z., Zhang Y. (2022). Ion migration in hybrid perovskites: Classifcation, identification, and manipulation. Nano Today.

[17] Yerezhep D., Omarova Z., Aldiyarov A., Shinbayeva A., Tokmoldin N. (2023). IR Spectroscopic Degradation Study of Thin Organometal Halide Perovskite Films. Molecules.

[18] Emelianov N.A., Ozerova V.V., Zhidkov I.S., Korchagin D.V., Shilov G.V., Litvinov A.L., Kurmaev E.Z., Frolova L.A., Aldoshin S.M., Troshin P.A. (2022). Nanoscale Visualization of Photodegradation Dynamics of MAPbI_3_ Perovskite Films. J. Phys. Chem. Lett..

[19] Ozerova V.V., Zhidkov I.S., Boldyreva A., Dremova N.N., Emelianov N.A., Shilov G.V., Frolova L.A., Kurmaev E.Z., Sukhorukov A.Y., Aldoshin S.M. (2021). Spectacular Enhancement of the Thermal and Photochemical Stability of MAPbI_3_ Perovskite Films Using Functionalized Tetraazaadamantane as a Molecular Modifier. Energies.

[20] Mester L., Govyadinov A.A., Hillenbrand R. (2022). High-fidelity nano-FTIR spectroscopy by on-pixel normalization of signal harmonics. Nanophotonics.

[21] Nečas D., Klapetek P. (2012). Gwyddion: An open-source software for SPM data analysis. Cent. Eur. J. Phys..

[22] Yuan Y., Chae J., Shao Y., Wang Q., Xiao Z., Centrone A., Huang J. (2015). Photovoltaic Switching Mechanism in Lateral Structure Hybrid Perovskite Solar Cells. Adv. Energy Mater..

[23] Govyadinov A.A., Amenabar I., Huth F., Scott Carney P., Hillenbrand R. (2013). Quantitative Measurement of Local Infrared Absorption and Dielectric Function with Tip-Enhanced Near-Field Microscopy. J. Phys. Chem. Lett..

[24] Park N.-G. (2015). Perovskite solar cells: An emerging photovoltaic technology. Mater. Today.

