# Supplementary material for: Direct Nanoscale Visualization of the Electric-Field-Induced Aging Dynamics of MAPbI3 Thin Films
Source: Materials (Basel). 2023 Jun 9;16(12):4277. doi: 10.3390/ma16124277 (PMC10303475; doi:10.3390/ma16124277)

Supporting information

Direct Nanoscale Visualization of the Electric-Field-Induced Aging Dynamics of $MAPbI_3$ thin films

Nikita A. Emelianov[1], Victoria V. Ozerova[1], Yuri S. Fedotov[2], Mikhail V. Zhidkov[1], Rasim R. Saifutyarov[3], Maria S. Malozovskaya[3], Mikhail S. Leshchev[1], Eugeniy V. Golosov[1], Lyubov A. Frolova[1], Pavel A. Troshin[4,5,1*]

1 Federal Research Center of Problems of Chemical Physics and Medicinal Chemistry, Russian Academy of Sciences, Academician Semenov Ave. 1, Chernogolovka 142432, Russia

2 Institute of Solid State Physics, Russian Academy of Sciences, Academician Osipyan Str. 2, 142432 Chernogolovka, Russia

3 National Research Centre "Kurchatov Institute," Moscow, 123182 Russia

4 Harbin Institute of Technology, 92 West Dazhi Street, Nan Gang District, Harbin 150001, China

5 Zhengzhou Research Institute of HIT, 26 Longyuan East 7th, Jinshui District, Zhengzhou 450000, China

* Correspondence: troshin2003@inbox.ru (P.A.T.)

Figure S1. SEM microscopy (a) and EDX mapping Si (b), Na (c), O (d) of $MAPbI_3$ channel after bias exposure for 16 days

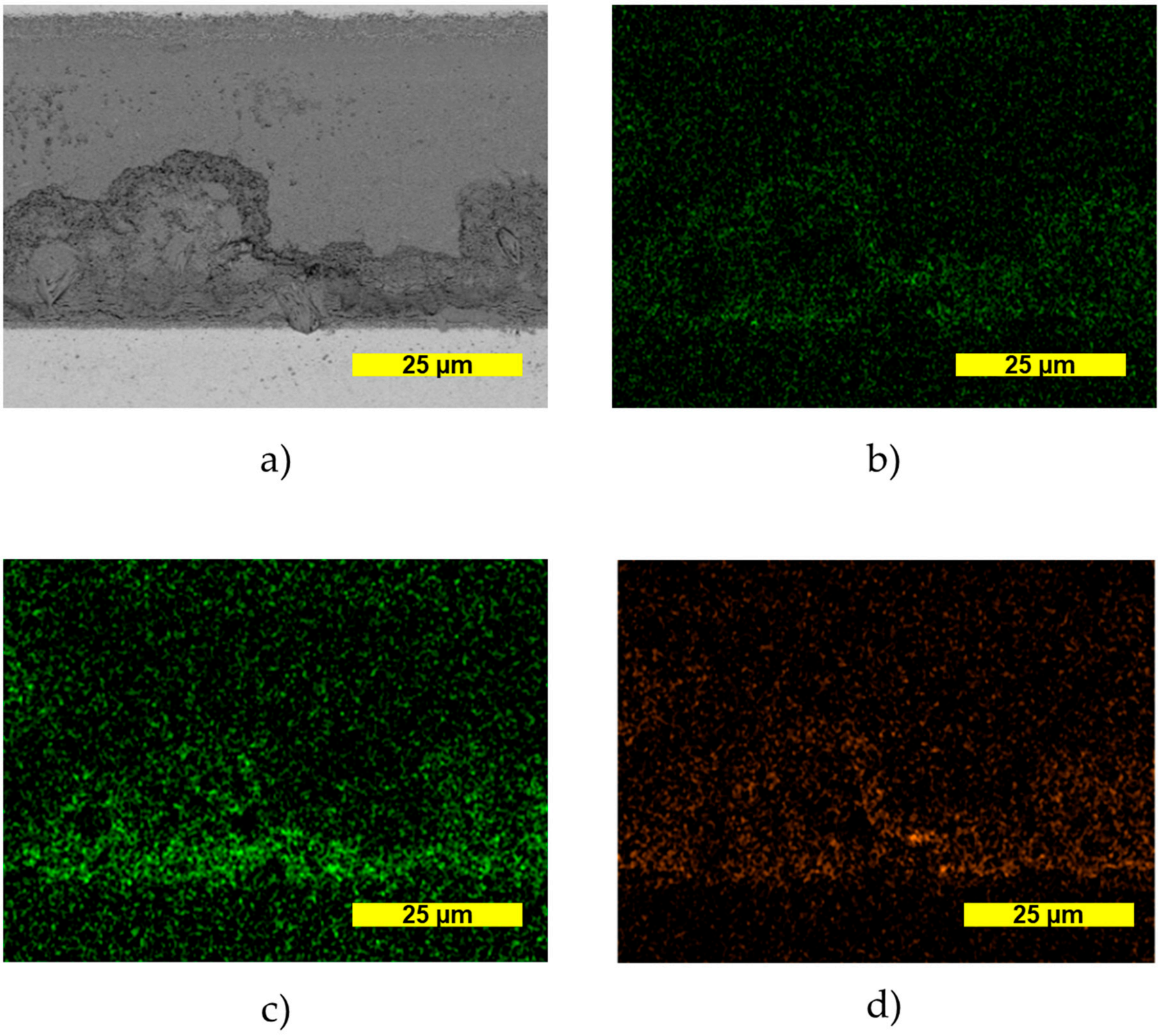

Supplement: Supplementary file 1 [file materials-16-04277-s001.zip › materials-2413611-supplementary.pdf]
